# Fruit size prediction of tomato cultivars using machine learning algorithms

**DOI:** 10.3389/fpls.2025.1516255

**Published:** 2025-01-29

**Authors:** Masaaki Takahashi, Yasushi Kawasaki, Hiroki Naito, Unseok Lee, Koichi Yoshi

**Affiliations:** ^1^ Research Center for Agricultural Robotics, National Agricultural and Food Research Organization (NARO), Tsukuba, Ibaraki, Japan; ^2^ Graduate School of Agricultural and Life Sciences, The University of Tokyo, Tokyo, Japan

**Keywords:** size prediction, fruit grade, machine learning, diameter, tomato

## Abstract

Early fruit size prediction in greenhouse tomato (*Solanum lycopersicum* L.) is crucial for growers managing cultivars to reduce the yield ratio of small-sized fruit and for stakeholders in the horticultural supply chain. We aimed to develop a method for early prediction of tomato fruit size at harvest with machine learning algorithm, and three machine learning models (Ridge Regression, Extra Tree Regrreion, CatBoost Regression) were compared using the PyCaret package for Python. For constructing the models, the fruit weight estimated from the fruit diameter obtained over time for each cumulative temperature after anthesis was used as explanatory variable and the fruit weight at harvest was used as objective variable. Datasets for two different prediction periods after anthesis of three tomato cultivars (“CF Momotaro York,” “Zayda,” and “Adventure.”) were used to develop tomato size prediction models, and their performance was evaluated. We also aimed to improve the model adding the average temperature during the prediction period as an explanatory variable. When the estimated fruit size data at cumulative temperatures of 200°C d, 300°C d, and 500°C d after anthesis were used as explanatory variables, the mean absolute percentage error (MAPE) was lowest for “Zayda,” a cultivar with stable fruit diameter, at 9.8% for Ridge Regression. When the estimated fruit size at cumulative temperatures of 300°C d, 500°C d, and 800°C d after anthesis were used as explanatory variables for Ridge Regression, the MAPE decreased for all cultivars: 10.1% for “CF Momotaro York,” 8.8% for “Zayda,” and 10.0% for “Adventure.” In addition, incorporating the average temperature during the fruit size prediction period as an explanatory variable slightly increased model performance. These results indicate that this method could effectively predict tomato size at harvest in three cultivars. If fruit diameter data acquisition could be automated or simplified, it would assist in cultivation management, such as tomato thinning.

## 1 Introduction

Fruit size and yield are crucial crop management considerations for horticultural fruit growers. These factors can vary based on weather conditions (Lötze and Bergh, 2004), crop load (Heuvelink, 1997; Naor et al., 2001), and responses to water and salt stress (Nuruddin et al., 2003; Itoh et al., 2020). Since dimensions, geometry, and fruit size are key determinations of fruit grade, for both growers and stakeholders in the horticultural supply chain, more precise predictions of these factors can enhance market value (Khojastehnazhand et al., 2010). Together with the total number of fruits, fruit size has a strong impact on yield estimation. Although technologies for predicting tomato yield using information on the growth environment and plant growth have been developed (Berrueta et al., 2020; Saito et al., 2020; Higashide, 2022), no methods for predicting tomato fruit size are currently available.

There are two lines of research into fruit size prediction. One uses a mathematical model based on environmental and crop growth data. Using this model, the average weekly error for cucumber fruit size was 6.6%, but at the end of the growing season, it was underestimated (Marcelis and Gijzen, 1998). Fruit size prediction can also be performed using mathematical models for peaches and tomatoes (Fishman and Génard, 2002; Liu et al., 2007). These techniques use preset parameters, which can lead to significant deviations from predictions when unexpected situations occur. The second is based on direct measurement methods with calipers and computer vision. Fruit size prediction at harvest based on measured dimensions over time during growth has also been done, but it is unsuitable for early-ripening apple cultivars (Zadravec et al., 2013). In greenhouses, counting fruits based on ripeness using visible images and estimating volume and surface area is easier (Zhao et al., 2016; Ziaratban et al., 2017; Gongal et al., 2018; Afonso et al., 2020; Lee et al., 2020; Ge et al., 2022). UAVs (Unmanned Aerial Vehicles) and robots could further improve yield prediction accuracy if the number of fruits in the entire field could be accurately quantified (Apolo-Apolo et al., 2020; Seo et al., 2021; Egi et al., 2022). By contrast, these techniques can only be used to predict the size of fruit close to harvest time. Given the antagonistic relationship between the size, composition, and number of fruits, predicting the size of fruits at an early stage is challenging even with current computer vision technology. Additionally, early fruit size prediction techniques are needed for artificial control, such as reducing the yield percentage of small-sized fruit.

Tomato fruits size is determined by cell number and cell size. Depending on the tomato cultivar, the division of pericarp cell progresses in a short period 12 to 25 days after anthesis (Bertin et al., 2009), and cell elongation continues until the start of fruit ripening (Giovannoni, 2004). Although most fruit volume increase occurs during cell elongation, final fruit size is highly correlated with the number of cells determined during early cell division (Bertin et al., 2009). After the middle stage of fruit enlargement, temperature strongly influenced the volume growth rate, which was lower at 14°C (low temperature) and 26°C (high temperature) compared to 18°C and 22°C, respectively (Adams, 2001). From the above, it is possible that the final size of harvested fruit can be predicted by two factors: the rate of volume increase and the temperature conditions, which are especially critical during the initial cell division phase of fruit enlargement and the middle growth stage. Fruit diameter has been widely used across various crops to estimate size and calculate the growth rate (Warrington et al., 1999,; Minchin et al., 2003; Tran et al., 2017). Using actual measured diameter data of fruits, fruit size can be estimated nondestructively over time and the rate of volume increase can be evaluated more accurately than by setting parameters in advance. It is also highly compatible with future use of computer vision-based technology.

In this study, we aimed to develop a technique for predicting the size of harvested fruit using the fruit size at the beginning and middle of the growth period, which we considered important for predicting harvested fruit size. To predict fruit size with high accuracy, we analyzed the data using various machine learning algorithms. We analyzed three tomato cultivars to identify the morphological characteristics those most adaptable to this prediction method and sought to enhance its precision by incorporating additional explanatory variables related to temperature.

## 2 Materials and methods

### 2.1 Plant materials and growth conditions

Plants were cultivated in a greenhouse (5.4 m width, 10.8 m length) at Tsukuba (36°01’N 140°05’E), Japan. Three tomato cultivars were used for this research: “CF momotaro York” (Takii & Co. Ltd., Kyoto, Japan), “Zayda,” and “Adventure” (Rijk Zwaan, De Lier, the Netherlands). The cultivation experiment was conducted between August 16, 2022, and April 27, 2023. Tomato plants were transplanted on coconut shell medium (Coco-bag; Toyotane Co., Ltd., Aichi, Japan) with a plant density of 3.75 stems m^-2^. Nine plants of each cultivar were surveyed up to about 15 or 16 trusses, with each truss limited to four fruit sets.

### 2.2 Tomato fruit data set acquisition method

**Figure 1:**
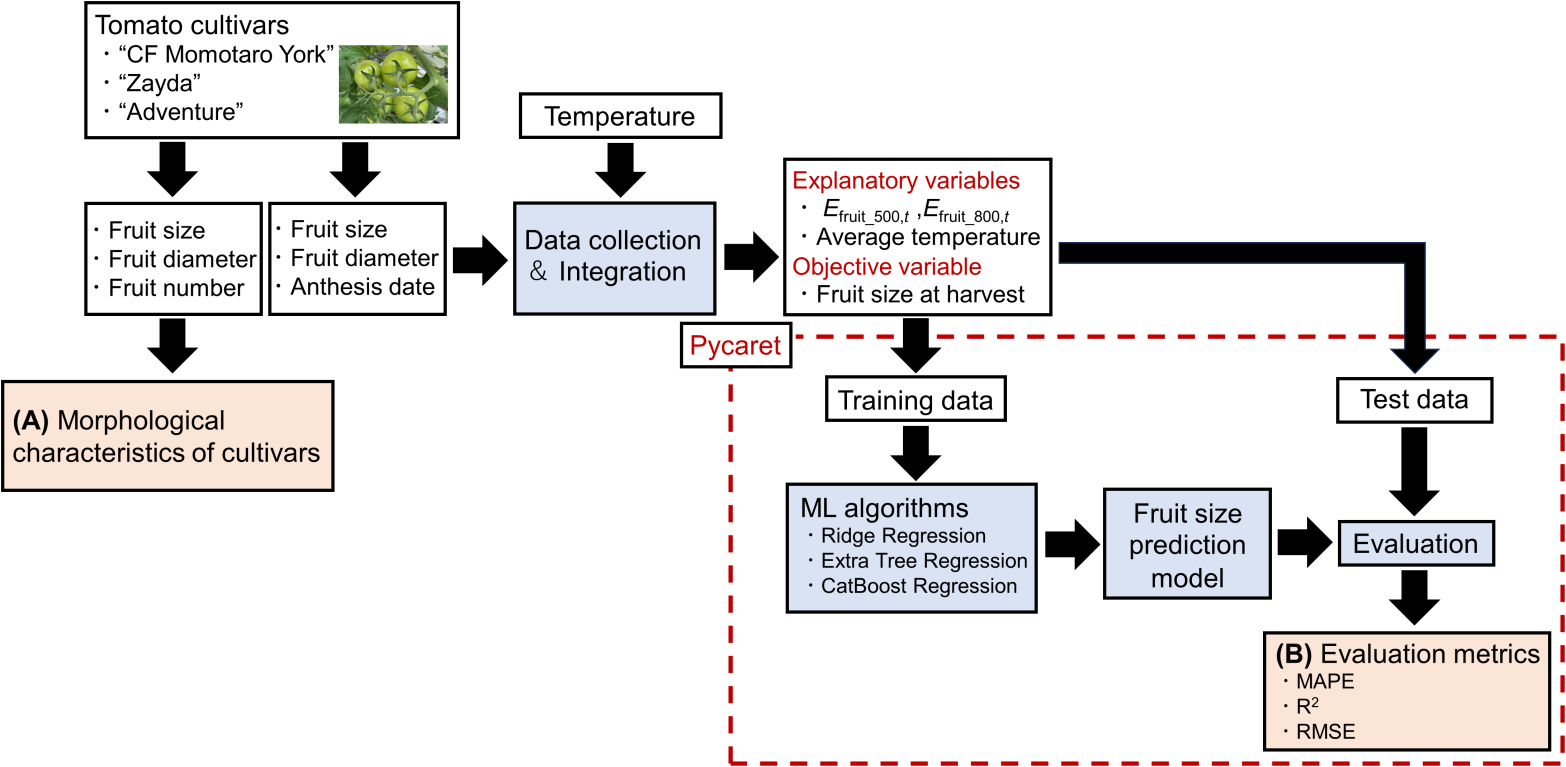
Overview of the proposal methodology. **(A)** Tomato size, fruit diameter and number of fruits were used in the analysis of morphological characteristics (Results Section 3.1). **(B)** The tomato fruit size prediction model was created using machine learning algorithms and evaluated (Results section 3.2, 3.3).

**Figure 2:**
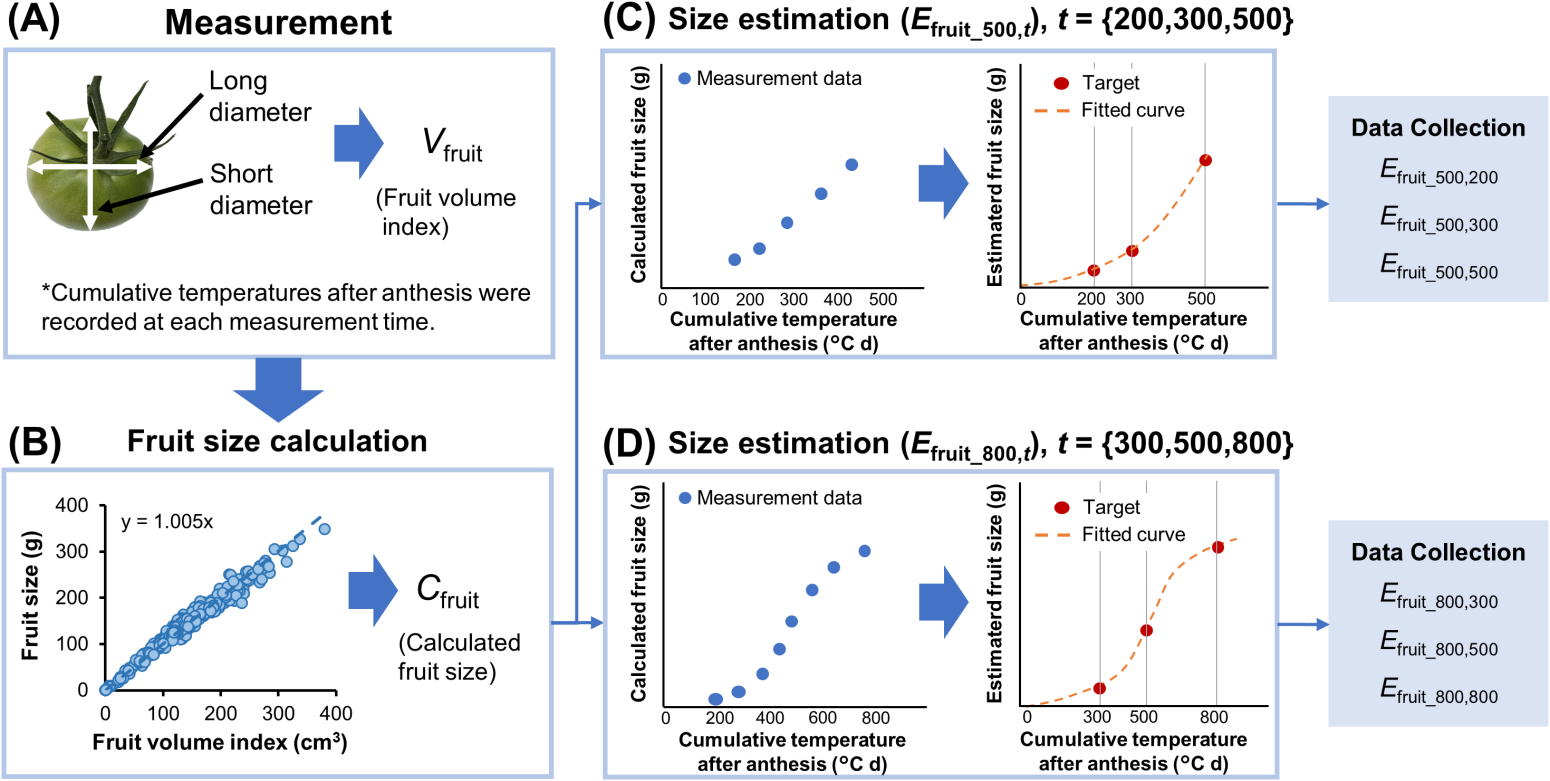
Proposed fruit size data collection and integration process. **(A)** Diameters of tomato fruit were measured at different cumulative temperature after anthesis of each flower, and *V*_fruit_ (Fruit volume index) was calculated. **(B)** The fruit density for each tomato cultivar was determined through regression equations based on *V*_fruit_ and actual fruit sizes, and *C*_fruit_ (the calculated fruit size) was calculated. **(C, D)** The estimated fruit size (*E*_fruit_500,t_, *E*_fruit_800,t_) for each cumulative temperature after anthesis was estimated from the fitted curve, and the data was collected.


Overview of the proposal methodology is shown in **Figure 1**. The first anthesis date of three tomato cultivars during the growing season was September 14, 2022, and they were continuously surveyed 2-3 times a week until March 6, 2023, for “CF momotaro York” and until February 27, 2023, for “Zayda,” and “Adventure.” The method of tomato data collection and integration is detailed in **Figure 2**. After fruit set, the long and short diameters of the fruit were measured once or twice a week and the data on the cumulative temperature after anthesis of each flower was recorded together (**Figure 2A**). These measurements continued until the fruit was harvested until April 27 for three cultivars. Tomatoes were assumed to be ellipsoids, and the fruit size of each cultivar was calculated from the long and short diameters using the following equation (Li et al., 2015).

(1) $$\begin{array}{c} V_{fruit}=\;\frac{4}{3}\;\pi \;\frac{l}{2}\;\;{\left(\frac{s}{2}\right)}^{2} \end{array}$$

(2) $$\begin{array}{c} C_{fruit}=\;V_{fruit}\;d \end{array}$$

where *V*_fruit_ represents the fruit volume index (cm^3^), *l* represents the long diameter (cm), and *s* represents the short diameter (cm), *C*_fruit_ represents the calculated fruit size (g), and *d* is the fruit density (g cm^−3^). The fruit density for each tomato cultivar was determined through regression equations based on *V*_fruit_ and actual fruit sizes after harvesting or thinning at various growth stages, 435, 547 and 449 fruits for “CF Momotaro York,” “Zayda,” and “Adventure,” respectively (**Figure 2B**). Using these equations, fruit size at each growth stage was calculated nondestructively from the diameters. As the date of anthesis differs for each tomato fruit and the temperature in the greenhouse is not constant, obtaining *C*_fruit_ at a specific cumulative temperature after anthesis is challenging. For each fruit, the *C*_fruit_ values at cumulative temperatures after anthesis were fitted to a third-order polynomial using Scientific Python (SciPy). A fitted curve (*r* ^2^ ≥ 0.94) was created using *C*_fruit_ up to cumulative temperature after anthesis<625°C d, and the estimated fruit size at 200, 300, and 500°C d was obtained (*E*_fruit_500,200_, *E*_fruit_500,300_, *E*_fruit_500,500_, **Figure 2C**). Similarly, a fitted curve (*r* ^2^ ≥ 0.95) was created using *C*_fruit_ up to cumulative temperature after anthesis<900°C d, and the estimated fruit size at 300, 500, and 800°C d was obtained (*E*_fruit_800,300_, *E*_fruit_800,500_, *E*_fruit_800,800_, **Figure 2D**). Since the pericarp cell division occurs 10–25 days after flowering and the cumulative temperature after anthesis is assumed to be<500°C d, fruit size (*E*_fruit_500,t_) was collected during this period. Additionally, 2 weeks before harvest, the cumulative temperature after anthesis was assumed to be ≥800°C d, and fruit size data (*E*_fruit_800,t_) was gathered to predict the harvest size by that time.

### 2.3 Model development

The data set was created using estimated fruit sizes (*E*_fruit_500,t_, *E*_fruit_800,t_) as explanatory variables and actual harvested fruit sizes as objective variables. For the estimated fruit size data sets (*E*_fruit_500,t_), 401, 516, and 417 fruits were used for “CF Momotaro York,” “Zayda,” and “Adventure,” respectively. For the estimated fruit size data sets (*E*_fruit_800,t_), 404, 524, and 421 fruits were used for “CF Momotaro York,” “Zayda,” and “Adventure,” respectively. The data analysis flow is shown in **Figure 1**. We used PyCaret 3.3, an open-source low code Python library that automates machine learning (AutoML) models (Moez, 2020). The library manages algorithms for regression and classification. The PyCaret library evaluates and compares these models based on specific metrics. Data were normalized using Z-score normalization and randomly divided into 80% for training and 20% for testing. Automated model selection was performed with PyCaret 3.3, where all existing regression models were trained and compared automatically based on the defined preprocessing pipeline for the dataset. From the recommended models, we selected and refined three: (1) Ridge Regression, (2) Extra Tree Regression, and (3) CatBoost Regression. Hyperparameters were optimized by repeated 10-fold cross-validation to maximize the determination coefficient (R^2^) in the training data with grid search (PyCaret’s default parameter). The model was then fitted to maximize the R^2^ for all training data. These analyses were conducted using PyCaret. Test data were used to verify prediction accuracy. To evaluate model performance, we used MAPE, R^2^, and root mean squared error (RMSE), which can be calculated as follows:


(3) $$\begin{array}{c} MAPE\;=\;\frac{100}{n}\sum_{i=1}^{n}\left|\frac{P_{i}-H_{i}}{H_{i}}\right| \end{array}$$

(4) $$\begin{array}{c} R^{2}\;=1-\;\frac{\sum_{i=1}^{n}{\left(P_{i}-H_{i}\right)}^{2}}{\sum_{i=1}^{n}{\left(P_{i}-\bar{H}\right)}^{2}} \end{array}$$

(5) $$\begin{array}{c} RMSE\;=\sqrt{\frac{1}{n}\;\sum_{i=1}^{n}{\left(P_{i}-H_{i}\right)}^{2}} \end{array}$$

where n represents the number of observations, *P_i_* represents the predicted tomato size, *H_i_* represents the harvested tomato size, and $\bar{H}$ represents the mean harvested fruit size.

### 2.4 Model improvement

To improve the prediction model, a new dataset was created that incorporated not only fruit size at each cumulative temperature after anthesis but also the average temperature during the prediction period as an additional explanatory variable. Using this dataset, the predictive model was developed with Ridge Regression. The model’s performance was evaluated by calculating the MAPE, R^2^ and RMSE following the same procedures described in Material and Methods Section 2.3.

## 3 Results

### 3.1 Morphological characteristics of the tomato cultivars

**Figure 3:**
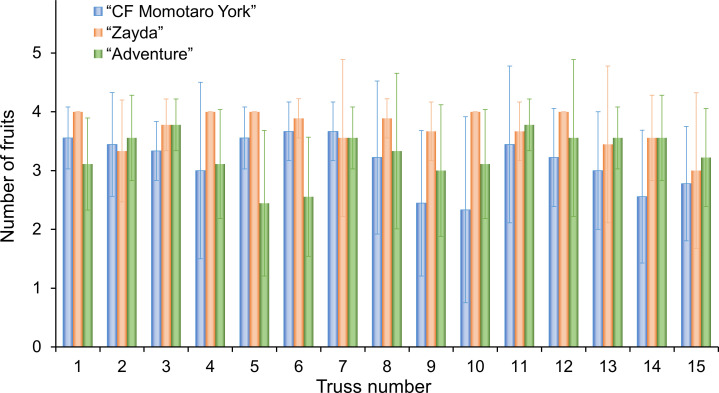
Number of fruits per truss for “CF momotaro York,” “Zayda,” and “Adventure.” Vertical bars indicate the SD of the means (n = 9).

**Figure 4:**
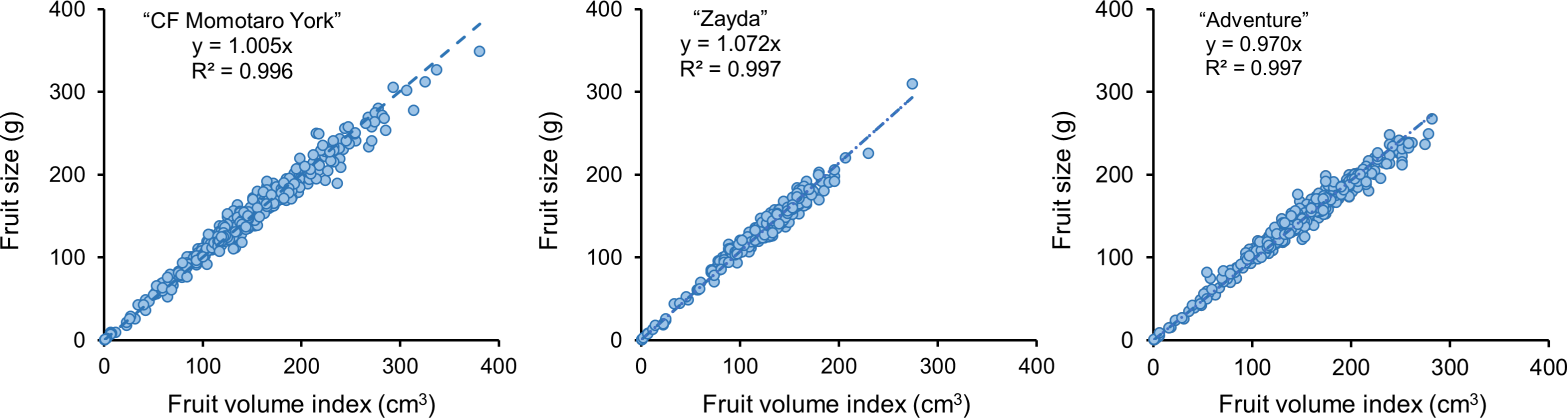
Relationship between fruit volume index (*V*_fruit_) and actual fruit size in “CF momotaro York” (n = 435), “Zayda” (n = 547), and “Adventure” (n = 449).

We examined the morphological characteristics of the fruits from each cultivar (**Table 1**). “CF Momotaro York” and “Adventure” had larger fruit size, long diameter and short diameter compared to “Zayda.” The standard deviation (SD) for fruit size, the ranking of cultivars from largest to smallest was “CF Momotaro York,” “Adventure,” and “Zayda.” The number of fruit sets per truss for each cultivar was also analyzed (**Figure 3**). “Zayda” showed particularly stable fruit set, with no fruit loss observed at nodes 4, 5, 10, and 12 across a survey of nine plants. The fruit volume index (*V*_fruit_) for each cultivar was calculated using the long and short diameters of the fruit (**Figure 4**). The results indicated that the calculated fruit densities (*d*) for “CF Momotaro York,” “Zayda,” and “Adventure” were 1.005, 1.072, and 0.970, respectively.

**Table 1:** Fruit size, long diameter, and short diameter of each tomato cultivar.

Cultivar	Fruit size (g) Mean ± SD	Long diameter (cm) Mean ± SD	Short diameter (cm) Mean ± SD
“CF Momotaro York”	154.3 ± 51.5a	6.7 ± 0.9a	6.4 ± 0.8a
“Zayda”	140.9 ± 27.4b	6.4 ± 0.5b	6.2 ± 0.5b
“Adventure”	152.0 ± 39.2a	6.7 ± 0.7a	6.6 ± 0.7a

Data are presented as the means ± SD. Different letters in a column indicate significant differences at the 1% level according to the Tukey–Kramer test.

### 3.2 Evaluation of prediction models

Estimated fruit size data (*E*_fruit_500,_ *_t_*, *E*_fruit_800,_ *_t_*) were used as explanatory variables to predict actual harvest fruit size through machine learning, utilizing three different regression models. The MAPE, R^2^, and RMSE of “CF Momotaro York,” “Zayda,” and “Adventure” are shown in **Table 2**, based on the predicted fruit size data. Ridge Regression consistently demonstrated stable and highly MAPE, R2, and RMSE for each cultivar. When *E*_fruit_500,_ *_t_* was used as the explanatory variable, “Zayda” had the lowest MAPE values, followed by “Adventure,” and “CF Momotaro York,” regardless of the regression model. When *E*_fruit_800,_ *_t_* was used, “Zayda” again had the lowest MAPE, with minimal differences between “CF Momotaro York” and “Adventure,” both showing MAPE values around 10%. These results indicate that prediction accuracy improves as the fruit develops and that the performance of the models varies by cultivar.

**Table 2:** Results of fruit size prediction at harvest.

Cultivar	Model	MAPE (%)		R^2^		RMSE	
		Explanatory variables^1^					
		*E*_fruit_500,_ *_t_*	*E*_fruit_800,_ *_t_*	*E*_fruit_500,_ *_t_*	*E*_fruit_800,_ *_t_*	*E*_fruit_500,_ *_t_*	*E*_fruit_800,_ *_t_*
“CF Momotaro York”	Ridge Regression	17.2	10.1	0.48	0.8	32.5	19.6
	Extra Tree Regrreion	17.1	9.7	0.51	0.81	31.9	19.3
	CatBoost Regression	17.0	10.0	0.44	0.78	33.8	20.6
“Zayda”	Ridge Regression	9.8	8.8	0.58	0.75	19.1	13.6
	Extra Tree Regrreion	10.0	8.9	0.43	0.71	22.4	14.8
	CatBoost Regression	10.3	8.5	0.42	0.74	22.5	14.1
“Adventure”	Ridge Regression	15.5	10.0	0.57	0.81	27.1	16.4
	Extra Tree Regrreion	16.5	10.3	0.44	0.77	30.9	18.0
	CatBoost Regression	16.7	9.6	0.48	0.81	26.7	16.7

^1^Explanatory variables are those used for fruit size predictions. “*E*_fruit_500,_ *_t_*” represents the estimated fruit size at cumulative temperatures after 200°C, 300°C and 500°C d anthesis were used an explanatory variable. “*E*_fruit_800,_ *_t_*” represents the estimated fruit size at cumulative temperatures after 300°C, 500°C and 800°C d anthesis was used as explanatory variable.

### 3.3 Improvement of prediction models

**Figure 5:**
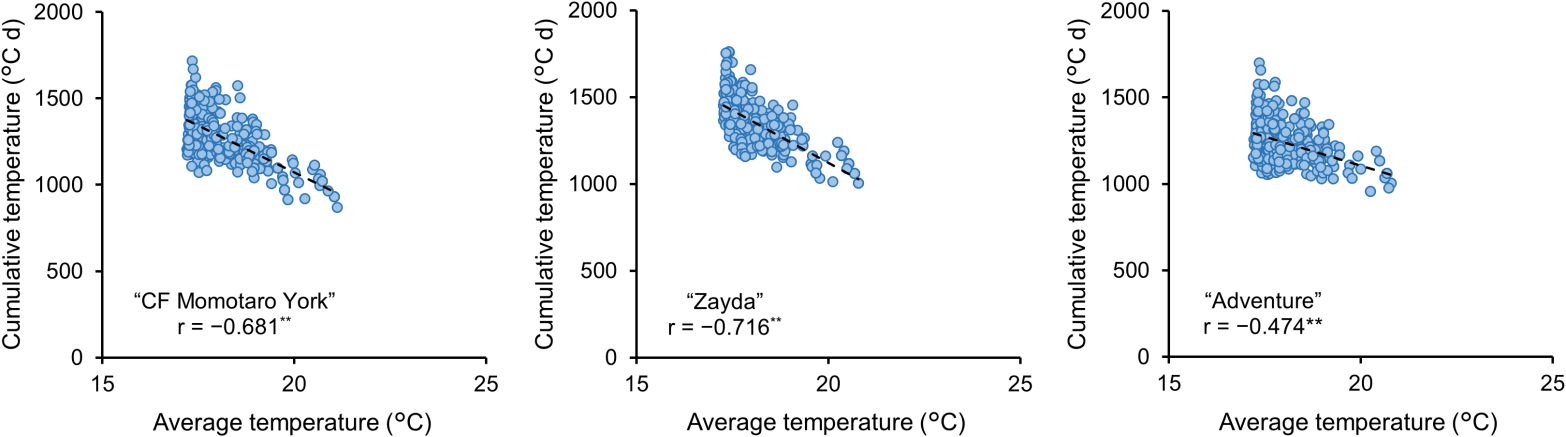
Relationship between average temperature and cumulative temperature from anthesis to harvest in “CF momotaro York” (n = 416), “Zayda” (n = 530), and “Adventure” (n = 431). **: significant negative correlation between average temperature and cumulative temperature (*P*< 0.01). Periods over which average temperature and cumulative temperature are calculated from anthesis to harvest date for each fruit.

We investigated the relationship between the average temperature and cumulative temperature from anthesis to harvest (**Figure 5**). The results indicated that for all cultivars, as the average temperature during the fruit growth period increased, the cumulative temperature until harvest decreased. The correlation coefficients were −0.681, −0.716, and −0.474 for “CF Momotaro York,” “Zayda,” and “Adventure,” respectively, with a *p*-value of less than 0.01 for all cultivars, indicating strong statistical significance. In an effort to improve the predictive model, we incorporated data showing that the time to harvest varies depending on the average temperature during fruit enlargement. By adding average temperature during the prediction period as an explanatory variable for fruit size prediction, the model’s performance improved for some cultivars and specific periods, however, the overall improvement was not substantial (**Table 3**).

**Table 3:** Results of fruit size prediction with the addition of average temperature as an explanatory variable.

Cultivar	Model	Average temperature^1^	MAPE (%)		R^2^		RMSE	
			Explanatory variables^2^					
			*E*_fruit_500,_ *_t_*	*E*_fruit_800,_ *_t_*	*E*_fruit_500,_ *_t_*	*E*_fruit_800,_ *_t_*	*E*_fruit_500,_ *_t_*	*E*_fruit_800,_ *_t_*
“CF Momotaro York”	Ridge Regression	+	17.2	9.8	0.50	0.82	32.1	19.0
		−	17.2	10.1	0.48	0.80	32.5	19.6
“Zayda”		+	9.4	7.6	0.63	0.81	17.9	12.0
		−	9.8	8.8	0.58	0.75	19.1	13.6
“Adventure”		+	14.8	10.0	0.59	0.81	26.3	16.3
		−	15.5	10.0	0.57	0.81	27.1	16.4

^1^Average temperature represents the period from anthesis to the recorded fruit diameter. “+” and “−” indicate whether the variable was used as an explanatory factor or not. ^2^Explanatory variables used for fruit size predictions. “*E*_fruit_500,_ *_t_*” represents the estimated fruit size at cumulative temperatures after 200°C, 300°C and 500°C d anthesis were used as explanatory variable. “*E*_fruit_800,_ *_t_*” represents the estimated fruit size at cumulative temperatures after 300°C, 500°C and 800°C d anthesis was used as explanatory variable.

## 4 Discussion

### 4.1 Morphological characteristics suitable for fruit size prediction

In this study, the morphological characteristics of the fruits of “CF Momotaro York” (a Japanese cultivar), “Zayda,” and “Adventure” (Dutch cultivars) were found to be differ from one another. Although the number of fruit sets per stem was limited to four in this experiment, “Zayda” exhibited the smallest fruit size. SD in fruit size was smallest for “Zayda,” followed by “Adventure,”and “CF Momotaro York.” “Zayda” showed the most stable fruit set (**Table 1**, **Figure 3**). The initial fruit growth rate and size differed depending on fruit load in a tomato truss (Bertin, 2005). These minimal fluctuations in the number of fruits suggest low variation in the morphological characteristics of fruits in “Zayda,” which may explain the high prediction accuracy and the machine learning model’s ability to perform well even at early stages of cumulative temperature after anthesis (**Tables 2**, **3**). When *E*_fruit_500,_ *_t_* was used as an explanatory variable, “Adventure” outperformed “CF Momotaro York” in terms of MAPE and RMSE in three machine learning algorithms, also indicating that variation in fruit size can influence prediction accuracy (**Table 2**). It is well-known that the number of days to harvest and fruit dry matter weight in tomatoes can vary depending on the growing season (Heuvelink, 1995b). In this study, the cumulative temperature from anthesis to harvest varied significantly with changes in the average temperature during this period (**Figure 5**), resulting in corresponding changes in fruit size (data not shown). This suggests that prediction accuracy could be further enhanced by selecting cultivars optimized for fruit size prediction and developing seasonal models that align with fruit size trends at harvest.

### 4.2 Improvement of the model by adding average temperature

Given that average temperature significantly affects the time to harvest, we aimed to enhance the accuracy of our prediction model by incorporating average temperature during the fruit size estimation as an explanatory variable. Previous research found that the influence of maximum fruit diameter was minimal within the stable temperature range typically maintained in commercial greenhouses (Tijskens et al., 2016). Earlier studies demonstrated that tomatoes grown at varying temperatures of 14°C, 18°C, 22°C, and 26°C experienced longer harvest times at lower temperatures, with smaller fruit sizes observed at both 14°C and 26°C (Adams, 2001). This suggests that different average temperatures can lead to varying fruit growth stages when cumulative temperatures of 500°C d and 800°C d after anthesis are reached. While incorporating temperature data improved the accuracy of certain predictions, the overall improvement was not statistically significant (**Table 3**). The average temperature during data collection should be recorded, as the cumulative temperature after anthesis is essential for making these predictions. Thus, it is recommended that this data be included in future datasets for operational use.

### 4.3 Use of predictive technologies

In Japan, tomatoes are graded based on their shape and size, but there is no unified standard for grading; it varies depending on the cultivar, growing region, and consumer preferences. Generally, consumers tend to prefer slightly larger tomatoes, while smaller ones are often considered unmarketable. To produce larger, high-value fruits, tomato cultivation involves limiting the number of fruits per plant (Cockshull and Ho, 1995; Heuvelink, 1995a). Early prediction of fruit size distribution can optimize crop management practices to reduce fruit load. With this in mind, statistical methods have been employed to develop predictive models for fruit size. For example, a model for kiwifruit was created to estimate fruit size based on fruit diameter measurements (Minchin et al., 2003). For effective crop management, predicting model for the size distribution of the harvested fruit have been developed for apples, pears and citrus (Lötze and Bergh, 2004; Tanimoto and Yoshida, 2024). Pruning and thinning can be used to manipulate the crop to achieve the desired size distribution at harvest (Guardiola and García-Luis, 2000). In this study, to estimate the size of individual tomato fruits and apply it to crop management, fruit size predictions were based on the estimated fruit size (*E*_fruit_500,_ *_t_*) at cumulative temperatures after anthesis, ranging from 200°C d to 500°C d. For three tomato cultivars, MAPE was below about 17%, which allowed predicting size distribution based on measurements during the early stage of fruit enlargement. To facilitate rapid information sharing with supply chain stakeholders, fruit size predictions were made based on the estimated fruit size (*E*_fruit_800,_ *_t_*) at cumulative temperatures after anthesis, ranging from 300°C d to 800°C d. Under the assumption of an average temperature of 18°C d, a cumulative temperature of 800°C d after anthesis would typically correspond to about two weeks before harvest in most growing seasons. When the average temperature drops below 18°C, however, the cumulative temperature required to reach harvest increases (**Figure 5**). Predicting fruit size at this later stage of accumulated temperature still allows for timely information sharing before shipment. Depending on the season, it may be necessary to adjust the amount of data over time for the explanatory variables to improve prediction accuracy.

### 4.4 Automation of data collection

In this study, fruit diameter data was collected using calipers. To apply this approach in practical production settings, fruit diameter data will need to be obtained through image analysis. Accurate sensing of flowering dates will also be crucial, as fruit diameter is closely linked to cumulative temperature after anthesis. These technologies, aimed at achieving high precision, are currently under development (Nyalala et al., 2019; Lee et al., 2022). Computer vision systems can be used to quantify increases in fruit diameter, length, and volume (Song et al., 2014; Mildenhall et al., 2021). In this study, the fruit size in each cultivar was calculated based on the long and short diameters, but predicting fruit size using just one diameter would facilitate the process. The coefficient of determination between harvested fruit size and long fruit diameter was 0.91, 0.92, and 0.94 for “CF Momotaro York,” “Zayda,” and “Adventure,” respectively (data not shown). Although using only long diameters provides less accuracy compared to using both long and short diameters, focusing on a single diameter measurement may simplify data collection using computer vision. Automatic measurement of fruit diameter offers the advantage of high-frequency data collection. Frequent measurements through computer vision could enable earlier predictions of fruit size at harvest, potentially even sooner than the cumulative temperature benchmarks used in this study, which would reduce the labor required for monitoring. This technology will allow growers to adjust thinning practices to ensure the proportion of small-sized fruits at harvest remains low. In the future, integrating automated fruit diameter data collection with fast, predictive crop management strategies will be key to improving production efficiency.

## 5 Conclusion

In this study, we proposed a method for predicting the tomato fruits size at harvest time by analyzing time-series data on the diameter of tomato fruits at cumulative temperatures after anthesis using machine learning algorithms. We developed a model that can be used for cultivation management, such as fruit thinning, using fruit diameter data from the early fruit growth period, and a model that can be used for information sharing with supply chain stakeholders, assuming a prediction two weeks before harvest. The MAPE for fruit size prediction of the three tomato cultivars ranged from 9.8% to 17.2% and from 8.5% to 10.3%, respectively, and these predictions could be used for tomato producers. The difference in accuracy between cultivars was related to the SD of fruit size and diameter, and the prediction accuracy was higher when used with cultivar that had less variation in individual fruits. By adding the average temperature during the fruit size prediction period as an explanatory variable, in addition to fruit size, the performance improved depending on the cultivar and period. In this study, fruit diameter was measured using a caliper, but in the future, we are planning to use computer vision to measure diameter more frequently in order to estimate harvest size even earlier than the reference value for cumulative temperature used in this study. This will enable tomato producers to adjust thinning work and improve production efficiency and profitability.

## Data Availability

The original contributions presented in the study are included in the article/supplementary material. Further inquiries can be directed to the corresponding author.
